# What recovery domains are important following a total knee replacement? A qualitative, interview-based study

**DOI:** 10.1136/bmjopen-2023-080795

**Published:** 2024-05-09

**Authors:** Chetan Khatri, Fatema Dhaif, David Ellard, Jeremy Neil Rodrigues, Martin Underwood, Paul Mitchell, Andrew Metcalfe

**Affiliations:** 1 Clinical Trials Unit, University of Warwick, Coventry, UK; 2 Trauma and Orthopaedics, University Hospitals Coventry and Warwickshire NHS Trust, Coventry, UK; 3 Health Economics Bristol, University of Bristol, Bristol, UK

**Keywords:** Knee, QUALITATIVE RESEARCH, ORTHOPAEDIC & TRAUMA SURGERY

## Abstract

**Abstract:**

**Objectives:**

To explore people’s views of recovery from total knee replacement (TKR) and which recovery domains they felt were important.

**Design:**

Semi-structured interviews exploring the views of individuals about to undergo or who have undergone TKR. A constant-comparative approach with thematic analysis was used to identify themes. The process of sampling, collecting data and analysis were continuous and iterative throughout the study, with interviews ceasing once thematic saturation was achieved.

**Setting:**

Tertiary care centre.

**Participants:**

A purposive sample was used to account for variables including pre, early or late postoperative status.

**Results:**

12 participants were interviewed, 4 who were preoperative, 4 early postoperative and 4 late postoperative. Themes of pain, function, fear of complications, awareness of the artificial knee joint and return to work were identified. Subthemes of balancing acute and chronic pain were identified.

**Conclusions:**

The results of this interview-based study identify pain and function, in particular mobility, that were universally important to those undergoing TKR. Surgeons should consider exploring these domains when taking informed consent to enhance shared decision-making. Researchers should consider these recovery domains when designing interventional studies.

**STRENGTHS AND LIMITATIONS OF THIS STUDY**

- Using qualitative methods (interviews), this study was able to gain a rich understanding of recovery domains, which may not be possible by quantitiative means.
- This study used a purposeful sampling of preoperative, early postoperative and late postoperative to provide a range of viewpoints.
- Interviews were conducted by two orthopaedic academic clinicians. Resultantly, inherent biases may have been introduced based on experience and attributes of the researchers. The interviews sought to reduce this by discussing preconceptions before interviews.
- This study provides insight into domains of recovery, such as pain and function, but is unable to provide quantitative or relative importance of individual domains.

## Background

 Total knee replacement (TKR) is an established treatment option for end-stage knee osteoarthritis. It can improve quality of life by alleviating pain and disability caused by osteoarthritis TKRs are a high-volume procedure, with over 100 000 being performed annually in the UK.^1 2^ This number is set to rise over the next decade.^3 4^ While largely a successful procedure, up to 20% of people are dissatisfied.^5 6^ This may be due to ongoing pain, disability or complications such as infection or loosening.^7^

Improvements to care, often through new technologies, are being introduced to continue to improve outcomes. The measurement for success is often quantitative, focusing on metrics such as prothesis failure and surgeons relying heavily on technical success.^8^ Surgeon-based rating scales can be biased, and they often compromise technical measures, such as infection, component alignment and prosthesis survivorship. This may result in a disparity between a surgeon’s and patient’s viewpoint on the outcome.^9^ When surgeons collect outcomes from their patients, in fear of disappointing, patients often undervalue their issues.^10 11^

Most outcome measurement is now conducted through patient-reported outcome measures (PROMs). They often ask questions related to pain and function to amalgamate into one summary score. While informative and an objective measure of improvement, they can be misleading where there may be opposing trends, particularly in instruments with more than one factor/dimension. For example, an individual may have persisting or deteriorating pain, but this may be masked by improvement in function, demonstrating an overall improvement. PROM instruments do not allow for a granular insight into recovery and are unable to distinguish or quantify how much a measured change in an individual domain is valued by an individual.

Attitudes about outcomes after a procedure are influenced by a complex interaction of personal views and by the shared experience of individuals in their close environment.^12^ To understand this, recovery domains must be placed in context, with the experience and expectations best understood through qualitative, rather than quantitative methods. Interview-based studies in hip and ankle fractures have explored preferences and therefore identified which outcomes are important to people.^13^,^15^ Understanding which aspects of recovery are valued by people after undergoing a knee replacement is needed for clinicians who seek to inform their patients appropriately and effectively at the time of consent. For researchers, it provides a greater understanding of the recovery experience for people undergoing knee replacement. By understanding the domains that are important to people, this work would ensure that important domains are collected as outcome measures in research studies and prioritises topics when designing new interventional studies.

Previous qualitative work within the context of TKR has explored the experiences of people with chronic pain after TKR, finding that it results in considerable distress, adaptation and acceptance of continuing pain.^16^ In an interview study of 17 people in Turkey, Savci and Bi̇li̇k explored the experiences of people undergoing TKR and defined three phases of the treatment journey, including the (1) decision-making phase, (2) the enduring phase and the (3) adaptation phase.^17^ These studies have either explored different phases of TKR recovery or recovery domains in subpopulations. In this paper, we sought to explore the importance of various recovery domains after a general population undergoing TKR.

## Research question

What is important to people when recovering from a TKR?

## Aims

To explore the experience of recovery following a TKR.

## Methods

### Study design

Semistructured interviews were conducted on a one-to-one basis. In light of the COVID-19 pandemic, patient partners recommended conducting interviews virtually using either telephone or video-conferencing software (Microsoft Teams). The study was conducted and reported in accordance with the Standards for Reporting Qualitative Research.^18^

A topic guide was created based on a systematic review,^19^ which identified relevant topics to cover ensuring the same basic issues were discussed.^20^ An introduction at the start of each interview included the name and role of the researcher and a brief explanation of the purpose of the interviews was given. A case vignette was presented at the start of each interview to contextualise the interviews. By using a short vignette, or a short story about a hypothetical person, researchers are able to gain information about the participants’ own set of beliefs. Through normalisation of the situation, they are able to encourage participants to reveal personal experiences they may otherwise not be comfortable in discussing.^21 22^ The specific wording of the topic guide was developed and checked with participant partners. The full topic guide is available in online supplemental file 1.

The interview started with fixed open-ended questions (box 1), which were presented to all participants at the start of the interview. For the preoperative population, these questions were structured around the ideas, concerns and expectation framework.^23^ In the postoperative population, this was the interview topic guide was developed iteratively after each interview to ensure previous topics were covered. These flexible questions were only posed if the topic wasn’t brought up by the participant in response to the open-ended questions. Flexibility with the use of open-ended questions ensured that participants were able to introduce new issues. Interviewers were trained in active passivity, so as not to disrupt the participants unless there was a significant deviation from the scope of the interview.^24^

Box 1 **Fixed questions used for interviews**

**Preoperative participants**.

- Can you tell me about the reasons you are considering a knee replacement?
- Is there anything you’re worried about?
- What are you hoping your operation will achieve?

**Postoperativeparticipants**.

- Can you tell me how your operation went?
- How has your recovery been?
- What have been the benefits to you since having the operation?
- Did you encounter any problems since you had the surgery?

### Eligibility criteria

Adults aged 18 or over who were waiting for (preoperative) or have had a TKR for primary osteoarthritis of the knee were included. Those undergoing unicompartmental knee replacement or revision TKR were excluded.

Participants were identified using preoperative waiting list and from postoperative clinics and initially approached via telephone. A purposive sample was identified to achieve maximum diversity. This was informed by operation status (before operation, early or late postoperation), consultant (attending), age, gender and ethnicity). Within the postoperative group, sampling also accounted for those who were early postoperative (up to 3 months) and those late postoperative (beyond 3 months, up to 2 years). This was chosen to limit recall bias.

### Recruitment

Potential participants were identified by an electronic database of people who had been assessed by a clinician and after discussion, agreed to go ahead with a TKR. The lead researcher (CK) contacted people from this ‘waiting list’, with a written patient information sheet. After informed, written consent, participants were entered into the study. Any individual who was under the care of any of the authors was not approached.

### Data collection and analysis

Two researchers (CK 31M and FGD 28F) conducted interviews to minimise the risk of bias introduced by just one researcher doing the interviews.^24^ Both researchers are orthopaedic registrars (residents) and this was disclosed to participants. Interviews were voice recorded and transcribed ad verbatim. These transcripts, alongside reflective notes produced after each interview, formed the raw data for analysis.

Data were analysed according to constant comparison using thematic analysis as an underlying theory.^25^,^27^ The process of sampling, collecting data and analysis were continuous and iterative throughout the study. Resultantly, there was no sample size, as this was determined by when no new themes emerged (data saturation).^28 29^ Data were coded using NVivo (QSR International).

Data analysis was completed using a six-step Framework devised by Braun and Clarke.^26^ An immersion (intuitive) analysis style was used.^30^ This included (1) transcripts being read and re-read to become familiar with data, (2) initial codes being generated and then (3) grouped into themes, (4) themes were reviewed and then (5) defined and named. Finally (6) a production of a report was created. Themes were produced through data. Their importance was not purely decided by frequency but also by judgement of the researchers to the relevance and insight it gave to the research question. During analysis, themes were refined as new transcripts were analysed. Interviews were stopped when no new ideas or thoughts emerged, and thematic saturation was reached in two consecutive interviews.

A minimum of 25% of interviews were coded by two researchers (CK and FGD) to enhance reporting and ensure saturation had been achieved. This was to ensure that any personal biases of the lead researcher were minimised. Any discrepancies were discussed with a senior author. Only once both researchers were satisfied saturation was achieved were interviews stopped.

### Patient and public involvement

Two patient representatives informed the design and conduct of this study. They reviewed participant facing materials such as the participant information sheet. They were consulted about the wording of questions in the topic guide to ensure they were easily understandable. They were consulted on format of interviews and suggested virtual interviews to minimise risk of SARS-CoV-2 transmission and the associated risks in a perioperative population.^31^ They reviewed the final manuscript and provided feedback to clarify interpretations.

## Results and discussion

### Participants

Of the 54 potential participants who were approached, 9 refused to take part (did not want to take part=4, no time=1, hard of hearing=2, prefer not to say=2). A total of 20 people provided informed consent with 12 participants completing interviews. The interview population had a mean age of 63 (range 51–78) with 7 (58%) females. Their care was managed by six different consultants (attendings). Four participants were preoperative, four were early postoperative and four were late postoperative. The participants demographic details are described in table 1 and are identified by a participant number (P1–P12). The age of the participants is presented in categorical manner to prevent compromising the anonymity of participants.

**Table 1:** Demographic details of participants

Participant number*	Sex	Age range	Laterality	Operative status	Length of interview (minutes)
01	Male	60–65	Left	Late postoperative	28
02	Male	60–65	Left	Early postoperative	39
03	Male	50–55	Right	Preoperative	36
04	Male	60–65	Right	Late postoperative	35
05	Female	60–65	Right	Late postoperative	25
06	Female	75–80	Right	Preoperative	26
07	Female	50–55	Left	Early postoperative	18
08	Female	50–55	Left	Early postoperative	18
09	Female	65–70	Right	Preoperative	21
10	Female	60–65	Left	Preoperative	35
11	Female	70–75	Right	Early postoperative	19
12	Male	75–80	Right	Late postoperative	33

* Participant numbers have been changed using a random number generator from original study identifier to protect participant anonymity. Therefore, participant 1 may not represent the first participant to be interviewed.

### Themes

**Figure 1:**
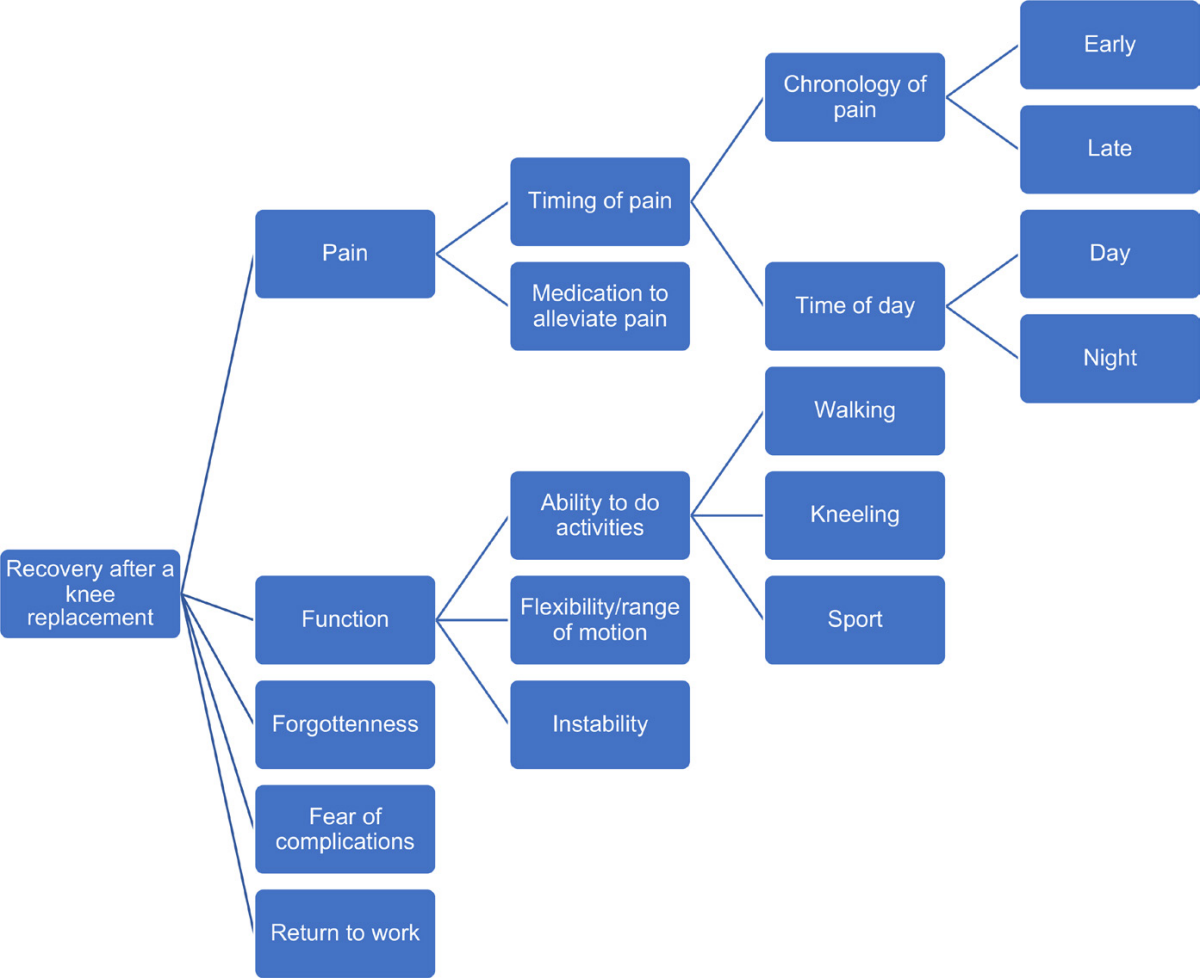
Code tree for themes and subthemes identified.

Five themes and nine subthemes emerged over the course of the interviews. The themes included pain, function, fear of complications, awareness of the artificial knee joint and return to work. These themes and subthemes are presented below with exemplar quotations. A full list of quotations is available in online supplemental file 2. A code tree is presented in figure 1.

### Pain

A common theme, mentioned by all participants, was the severity of pain. Three distinct subthemes emerged from this and are presented below.

#### Pain subtheme: early versus late pain

Many participants expected to eradicate their pain by having a knee replacement. However, participants were surprised by the amount of pain they were in immediately postoperatively. This pain was more than they expected and caused significant distress. As participants were often discharged and then suffer a substantial amount of pain, a feeling of abandonment was highlighted by a participant.

P5 (61F LATE POST-OPERATIVE): It was really bad when I got home. Thing is, you get the morphine and all that in the hospital, but you don’t get much of it to go home with. And then I didn’t know whether it was normal … or who I could go get some tablets from. You felt like you were all alone.

For one participant, the previous experience of intense postoperative pain meant she was reluctant to undergo a TKR on the contralateral knee. Some balanced and traded the higher postoperative pain in the hope they would have no pain in the long term. They stated they hoped to have no pain by the 1-year mark.

P5 (61F LATE POST-OPERATIVE): I don't want long term pain. No, I'd rather have that short term pain.

P2 (65M EARLY POST-OPERATIVE): Yes. Yeah, very bad. Yeah, I would say I would say 9 out of 10. Yeah. Really, really bad, really bad. It was when our first for the first three or four, first three or four days: really bad. I don't mind telling you that it did bring tears to my eyes when I came back from the theatre. It was absolutely excruciating. The pain was, yeah, it was a lot. A lot of pain. I didn't experience that pain ever. No, it really was a lot of pain.

#### Pain subtheme: daytime versus night-time pain

Participants who were preoperative discussed how night-time pain was causing them distress. In addition, participants who were postoperative reflected that night-time pain used to be an issue. They discussed how rolling in bed would elicit pain and subsequently disturb their sleep. However, their perception changed after knee replacement, the difference between daytime and night-time pain was not something participants stated caused them an issue.

P5 (61F LATE POST-OPERATIVE): It would just be there. It was just there. Yeah, I couldn’t say what you know any easy stuff with painkillers, but. It just was always there, you know, whether it went to a 10 or then dropped back a bit, but the pain, the pain was always there. It didn’t matter if it was at night or whatever.

P12 (78M LATE POST-OPERATIVE): No … as long as I got comfortable in bed, I didn’t feel the pain. I slept well. It used to be problem before I had the operation, but not really after.

#### Pain subtheme: medication to relieve pain

Two participants highlighted their aversion to having to take tablets to relieve pain. They believed a good effect of having a knee replacement would be to reduce their medication burden. In addition, they also saw the value of removing side effects such as constipation.

P6 (78F PRE-OPERATIVE): I suppose it’s me. My tummy doesn’t accept a lot of the painkillers very easily, and that was my problem, particularly with the right knee. The let’s say that the painkillers upset my tummy, so I had to. I did put up with more pain in the right one.

### Function

While function may be expected to be a desired recovery domain, participants described distinct domains with six subthemes emerged from the data. Of these, ability to walk was deemed the most important, being highlighted across the diverse population that was sampled. This subtheme overlapped with other subthemes such as return to work, recreational and activities of daily living emerged. Participants recognised a factor of interdependence where the ability to walk would also facilitate other domains.

#### Function subtheme: ability to walk

A consistent theme throughout the participants was their ability to walk. Reduced mobility was a common disability that participants suffered from which was the motivating factor to undergo an operation.

This was highlighted as importance for a variety of reasons. First, it was seen as essential to daily activities. Second, it was viewed as a leisure activity, to be shared with loved ones and for those participants who had not yet undergone surgery this was an aspect of their family life they felt they were missing out on. Third, it was seen important for exercise and health, both to be able to participate in their chosen sport but also as a substitute for those unable to join in sports. Finally, it was also essential for work for some participants, who were employed in active job roles.

P2 (65M EARLY POST-OPERATIVE): I love my walks. And when before I had the operation, we went me and my wife, we went away and we were walking down to the down the beach. I just couldn't walk no further. So I sat down. I sat on a bench and my wife kept on walking down the sea front. And then that really that really hit me. That did that to say that I've got to get this sorted out now. I’d just let my wife walk on her own and not with her. That’s when I decided I needed the operation.

P3 (53M PRE-OPERATIVE): I’ve missed out on afternoon walks or, you know, playing with, you know, nephews or nieces.

P4 (60M LATE POST-OPERATIVE): Very important because otherwise I feel unwell. I mean, I don’t get an awful lot of other exercise. That’s just because I can’t. I mean, I’m 61 now and I'd haven’t turned it to do any sport at for a number of years, to be quite honest with you. But walking obviously is important.

#### Function subtheme: ability to kneel

Participants highlighted their inability to kneel, which led to restrictions in their lives. For a few, it prevented work which required them to kneel. Resultantly they adapted by using knee pads or cushions to facilitate working. For another participant, it resulted in them changing their job role while simultaneously hoping that the knee replacement would allow them to return to their previous job role by being able to kneel.

Some participants highlighted that they were unable to kneel preoperatively as a result of their arthritis. They felt that even after the operation, they would not require to kneel for any activity. A few mentioned they compensated for this by modifying housework. Some participants hadn’t tried to kneel as they believed they were not supposed to, as this could damage to their knee prothesis. Due to a culmination of preoperative activity modification and postoperative expectations, most participants felt kneeling was not important to them.

P4 (60M LATE POST-OPERATIVE): I don’t. I haven't really tried it. I don’t think it’s the best idea to be doing that. Is it really? I don’t know. I haven’t really looked into it, but I haven't really needed.

I can function quite happily without I got. I'm gonna say I could function quite happily without kneeling down.

#### Function subtheme: normal activities in the home

Activities around the home were given differential importance. Going up and down the stairs was deemed to be important for those it was relevant to, as it allowed them a ‘normal’ way of living, with their bedroom upstairs. In addition, rising from sitting was deemed important, with a participant elaborating saying this enabled them to use the toilet normally.

Other tasks, such as chores around the house and gardening, were deemed less important. Participants often gave examples of either friends, family or carers taking over these tasks, which may mean their importance is diminished as these are not essential or personal tasks to many.

P4 (60M LATE POST-OPERATIVE): Things like sitting rising from an armchair and going into an armchair. Even the toilet. Excuse me but going to the toilets a lot more going to win now because it used to be I used to hold on to things to lower myself like you know. But now I can just go normally. So yeah, that has improved as well. So, I'm pleased about that, and it is important.

#### Function subtheme: flexibility and range of motion of knee joint

Participants discussed how stiffness and limitation on how much they could flex their knee joint impacted their life. Due to stiffness in the knee joint, they were unable to complete day to day movements, such as going up and down stairs. Additional movements like picking something from the floor were restricted by them being unable to bend their knee. In the initial stages after their operation, they described how postoperative swelling prevented them being able to move their knee effectively resulting in disability.

P10 (63F PRE-OPERATIVE): But because your leg is so swollen you really can’t bend it. And so you kind of that pushing into bends and trying to get it to bend. But you there’s no way it’s gonna bend because there’s just too much fluid in it. And so the you do feel a little bit abandoned a little bit on your own afterwards.

P1 (62M LATE POST-OPERATIVE): Well, it were locked in to not much more than a degree. So the very awkward kneeling down things to pick stuff up off the floor.

#### Function subtheme: instability

A participant described a feeling of instability in her knee as a result of osteoarthritis. This was a motivating factor for her to get a knee replacement. She described postoperatively that her knee replacement gave her stability which was the greatest reason for satisfaction of her procedure.

P5 (61F LATE POST-OPERATIVE): It would just seem to slide. I don’t know if you know what I mean? Sort of the top joint on the bottom joint just seemed to slide and it would be very unstable. And I felt that when I was walking, it was wobbling and moving inside the knee and it just would slip off, felt like it would slip off. …

(After the operation) I love my new knee. It was just stable. It didn’t wobble anywhere. It was just the next day was just great. So different. The best part of the new knee.

#### Function subtheme: sport

Some participants were able to play sport before their operation, for them, continuing their favourite sport after receiving a TKR was of great importance.

P2 (65M EARLY POST-OPERATIVE): Not really. Well, you spray played golf and not golfer snooker and bowls and that sort of thing. But I I’m I’ve got back to them yet because I just can’t stand up on both my legs properly for balance wise. So but yeah, that’s another goal. I want to get too.

Interviewer: How would you feel if you wouldn’t be able to go back to doing any sport after your new replacement?

P2 (65M EARLY POST-OPERATIVE): Ohh uh, I wouldn’t be very happy.

Others were not able to before the procedure, but either looked to be able to return back to sport or modified their expectation by either changing their preferred sport or having aids to make it easier to participate. For a substantial number, they could not participate in sports before the procedure and did not expect to be able to return.

P4 (60M LATE POST-OPERATIVE): Was playing sport important to me? Yes, it was in years gone by, but not now. Not now. Not an age now. No, I'm not playing sport. …

### Fear of complications

Most participants did not have apprehensions about complications such as bleeding or infection. They rationalised this by the low chance of them occurring, comparing them to the risk of day-to-day tasks such as having an accident while out of their home. They further weighed this by acknowledging the precautions the surgical team took to reduce the risks, such as strict aseptic procedures to reduce the risk of infection. They felt the potential benefits of the procedure outweighed what they perceived to be low risks.

P3 (53M PRE-OPERATIVE): Well, bleeding’s always a risk. Whenever you do surgery. But then, you know, I think that’s probably a less of a risk inside and because it is a controlled environment, everyone knows what’s going on. You gotta look at the stats, right. And what’s the likelihood of that, one in 100,000?

Participants deemed the risk of infection to be important if this led to a second procedure, or a revision procedure. Indeed, the risk of a revision or second procedure for any cause was a major worry for participants. They worried about the additional exposure to risk of complications carried by a second procedure. Importantly, participants emphasised that a reoperation would restart their recovery, linking this to the high levels of unexpected pain they endured after their first procedure.

P2 (65M EARLY POST-OPERATIVE): Well, yeah, you know, I’ve got friends while I had to go back in and add reoperations and that they didn’t like it at all. Then it’s really put them back, you know, in weeks and more pain and what have you. And I just did not want to go down that road. So I tried to do everything because the doctors and the nurses and everybody told me to do and no fingers crossed things have worked out quite nicely for me just apart from them to go back in to have it and. And let’s say that put a chill down my neck and I thought ohh dear. That’s something I don’t want. And even he (my friend) said the same. You know, he (participants friend) was dreading them out him up again to redo what was wrong. Like I don’t wanna go back where I've just came from.

Participants who were either diagnosed with or had a suspected blood clot, discussed the distress this caused them. They described the worry while waiting for the diagnosis to be confirmed and how this would affect their knee replacement. As a symptom they were experiencing was often pain, they described the pain as causing distress and worry.

P4 (60M LATE POST-OPERATIVE): Now it was it was suspected DVT*. It wasn’t actually confirmed as DVT as such, but I did actually undergo treatment for it. … It was a concern at the time because it was very, very painful. My, my leg was very well, very sore, I couldn’t move properly cause of the pain, you know. And did the pain mean something was wrong in my knee joint?

*DVT in the quote is in reference to deep vein thrombosis.

A participant attributed the risk of complications or dissatisfaction due to the surgeon performing the procedure. Therefore, they perceived the reputation of the surgeon to be of great importance.

P10 (63F PRE-OPERATIVE): I think this comes down to having trust in your surgeon, doesn’t it? I mean, I'm kind of well aware that, you know, I think it’s 80% of knees had done very, very well and are successful and 20% aren’t and I wouldn’t be at all surprised. That’s largely to do with the surgeon or whatever. … So his reputation was important to me.

### Awareness of artificial knee joint

A theme that emerged was the importance of being aware, or conversely being able to ‘forget’ that the participants had an artificial knee joint. There was a wish for them to want to think the implant was a part of their body. Notably, there were some participants who were unable to understand the concept of being able to forget about having an artificial knee joint. Participants explained this as wanting to be aware of their joint, to modify their activities with a view to prevent damage to their prosthetic joint.

P4 (60M LATE POST-OPERATIVE): Yeah, it’s important to be aware of it. It’s getting to the stage where it feel it’s beginning to feel like part of me, if you like. As time goes on, it’s beginning to feel more part of me, which I'm pleased about before.

### Return to work

For a substantial population, they were retired and therefore their knee replacement had no influence. For those that did work, the osteoarthritis in their knee had resulted in modifications to their work leading to a reduction in physical demanding work (increased desk work). In some cases, they hoped that after the operation they could return to a higher or predisease level of work. Some felt a delay in receiving their operation would reduce their chance of recovering or attaining a pre-disease level of activity.

P8 (51F EARLY POST-OPERATIVE): I’m a police officer, so I was removed off sort of uniform duty to desk duties and. Just couldn’t walk more than sort of more than 10 minutes without severe pain in the knee, so it got to the point where it was getting more desperate than anything. I needed my knee to be sorted to get back on the beat (active patrol).

P5 (61F LATE POST-OPERATIVE): Yeah, I mean, I wanted to go back straight away. Yes, as I say, I’m mindful. So I didn’t go in the playground with the children because I felt if I didn’t want to be knocked into or knocked over when they run. … Absolutely would not want to change my job

## Discussion

In this study, we interviewed 12 participants to establish their views on what is important to people when recovering from a TKR. We interviewed a purposeful sample of participants who were preoperative, early postoperative (up to 6 weeks) and late postoperative (up to 2 years). Using thematic analysis, we identified five themes which included (1) pain, (2) function, (3) fear of complications, (4) awareness of artificial knee joint and (5) return to work.

### Pain

Survey-based research has shown postoperative pain (and mobility) to be of the most prominent concerns.^32 33^ A systematic review and meta-synthesis of 28 qualitative studies demonstrated patients did not understand what the normal resolution of pain should be postoperatively.^34^ Clinicians should be aware of this, in ensuring effective pain management strategies. This must extend to the immediate postdischarge phase, including strategies to anticipate changing analgesic requirements.

For some participants in this study, this early postoperative pain was traded with hope for no pain at the 1 year mark. People often underreport their pain, despite chronic pain being a cause of considerable distress.^16^ To help identify these issues, clinicians may consider the use of instruments that not only measure the severity of pain, but its impact on the individual. An example of this would be the Intermittent and Constant Osteoarthritis Pain Questionnaire, which explores frustration, annoyance and impact on quality of sleep.^35^

Commonly used PROM instruments such as the Oxford Knee Score (OKS) and Forgotten Joint Score (FJS) include a variety of items assessing daytime and night-time pain.^36^,^38^ While important in the preoperative state, this study reveals it is not an issue in the postoperative phase. The content validity for these PROMS should be reconsidered for the postoperative population.

### Function

Being able to walk was a large motivating factor for individuals to undergo an operation and has been cited it as the most common reason to undergo surgery within the literature.^39^ It is assessed in the OKS and FJS, examples include ‘walking for 15 min, climbing stairs and doing housework/gardening’.^36^,^38^ Furthermore, the ability to walk has been shown to show the greatest improvement in health-related quality of life.^40^ In an interview study of 25 participant, Woolhead *et al* found participants to highlight inability to walk as an issue after TKR.^41^ The reasons behind how this was perceived was not fully developed by Woolhead *et al*. This study may provide insight into this. The reasons for were not only for activities of daily living but for recreation and socialising. Within the preoperative population, a survey-based study found questions such as ‘how mobile will I be?’ and ‘when will I be able to walk normally again?’ in the top four highest rated questions, giving further weight to its importance to people.^32^

The ability to kneel is another item frequently assessed in PROM instruments such as the OKS^37 38^ and Knee and Osteoarthritis Outcome Score (KOOS).^42^ This study identified that kneeling is not important to all participants. Many had modified their activities preoperatively because of pain caused by osteoarthritis in their knee. They therefore did not require the ability to kneel postoperatively. Nevertheless, for some individuals, the ability to kneel was crucial for their work. It is known that TKR surgery often fails to meet the expectation of kneeling for those who require it.^43^ Survey-based research exploring the OKS and KOOS has reported kneeling being important to as low as 32.7%–52%, respectively, agreeing with this study.^44 45^

Several participants identified that the ability to return to playing their favourite sport was not important recovery domain for them. This was due to a combination of factors: (1) their age and resulting inability to participate due to other health conditions and (2) the disability brought on from knee osteoarthritis which had resulted them withdrawing from sporting activities before the operation, with no desire to return. This may, in part, explain the high rate of non-completion of this item within instruments such as the FJS.^46^

### Fear of complications

This study found a fear of complications to be a worry to participants, particularly those that require a second operation. Other studies have recognised the worry and fear a second operation carries.^47^ This apprehension is important, as postsurgical complications have been identified as one of the top three reasons for dissatisfaction after TKR.^48^ Specifically, complications requiring readmission can lead to greater dissatisfaction.^5^ Other qualitative studies have identified fear of complications as a key decision-making marker when considering TKR.^12^ Clinicians should be aware of the fear involved with a return to theatre, empathising and being sensitive to what is a distressing time.

### Awareness of artificial knee joint

The FJS is based on the concept of being awareness of the artificial joint as an underlying unidimensional trait.^36^ While this may represent a combination of pain and function, the instrument measures how aware people are of their artificial joint while doing different activities. Within this study, however, some individuals were unable to understand the concept of being able to forget their artificial knee joint. This was justified by some as wanting to be aware, to modify their activities with a view to prevent damage to their prosthetic joint. In other studies, those who had residual symptoms, whether that be pain, restrictions on certain movements, viewed their knee joint as an ‘alien body part’.^49^ Indeed, for those who had complete resolution of symptoms, a goal was to have a ‘silent’ knee joint, allowing them to continue living their lives before the onset of osteoarthritis. Resultantly, they felt the goal of striving for a ‘silent’ knee was the ultimate goal for their participants.^49^ However, those with pain and restrictions in mobility were unable to forget their knee joint, which may represent the position for individuals in this study.

### Return to work

Previous survey-based work has identified around between 75% and 90% of people who are of working age (<65 years) are employed prior to TKR.^50 51^ Of those aged <50, all were found to go back to work after TKR while only 24% of those aged 60–65 did. While this aim to return to work is an aim for the younger, more active and higher demand participants, it is not applicable for more elderly population who undergo TKR. Qualitative work exploring experiences in returning to work in a younger (<60 years age) also identified delayed surgical intervention as a theme.^52^ This is particularly important in a younger, high-demand profession population.

### Strengths and limitations

Interviews represent an opportunity to gain a richer and individualised insight into individuals’ preferences and opinions. This gives understanding of individual recovery domains, which incorporate range of biological, psychological, emotional and contextual influences. Meanwhile, PROM instruments are constrained by the questions within them but can describe a subpopulation in a standardised and repeatable manner. In this study, by using interviews, we were able to gain a deeper understanding of themes such as pain, which may not otherwise be understood if using PROM instruments alone. We were able to consider possible reasons why certain items, such as sport, may have high missingness in PROM instruments (FJS).

Interviews were conducted per participant at one time point. Therefore, this study is unable to explore individual level change in perception of the importance of recovery attributes. However, purposeful sampling across balanced groups of preoperative, early and late postoperative allowed this study to gain a valuable insight into a broad range of recovery after TKR.

This study is limited to drawing its sample population from one geographic area. With research suggesting inequity in the provision and outcomes of TKRs across the UK,^53^ these findings may not be transferable to the wider population. Nevertheless, the study demographics are comparable to the population of people considering of have received a TKR.^54 55^

We noted that three interviews were less than 20 min in length (table 1). We reviewed all three transcripts with critical reflectivity to ensure that participants were able to fully develop answers. We note all three participants were early postoperative and stated that they were in a greater than expected amount of pain. While we did not collect any objective measures of pain (such as a Visual Analogue Score), we postulate whether this pain may have curtailed answers, and therefore, length of the interview.

Interviews were conducted by two researchers who were both academic trainees in orthopaedic surgery. As a result, a bias may have been brought in by the questions that were asked or by the responses received. To minimise this, the interviewers introduced themselves as researchers rather than surgeons. Preconceptions were discussed between researchers to minimise this introducing bias. With subsequent manuscripts coded independently, the coding structure was audited by the senior supervisory team.^24^ Furthermore, it ensured true thematic saturation was reached, rather than just perceived to have been by one researcher. It allowed supplementing and contesting individual researchers opinions enhancing reflexivity.^24^

While this study explores both domains of interest, it does not give preferential weight of one domain over the other. It cannot answer the question of ‘is short-term pain or long-term pain more important?’ in a quantifiable manner. It is also unable to determine whether different sub populations (such as those preoperative or postoperative) have different preferences in recovery after TKR. A health preferencing study, such a discrete choice experiment, would allow us to use information gathered from this study to generate a hierarchy of domains of which outcomes are most important to people.^56^

### Implication for clinicians and researchers

Clinicians should consider the limitations that PROM instruments provide in measuring success. While a useful guiding tool, they should be supplemented with knowledge from qualitative work on issues that matter to the people they care for. Using sensitive questioning, ascertainment of whether there is residual pain or immobility can give further insight into the perceived outcome of an individual.

Furthermore, clinicians should take care to explain and give reasonable expectations for pain and function, in particular mobility. Meta-synthesis of qualitative research has demonstrated that this information is not always conveyed.^34^ Better communication and individual assessment of needs has been suggested to improve care.^11 57^ This improved communication mostly consists of preoperative education, which has shown to improve surgical outcomes.^58^ This involves not only the consultation with the clinician but further supplemented by educational resources, tailored to the individual by its availability in numerous formations.^59^ High levels of early postoperative pain caused distress for participants in this study, modulating this with preoperative expectations has been shown to improve the ability to manage increased pain levels.^60^

When considering which outcome measures to use in interventional studies, particular care should be given to those that investigate domains of interest captured in this review. Both early and late postoperative pain should be captured. When PROM instruments contain multiple underlying traits (eg, can be split into pain and function), these should be considered separately.

## Conclusion

This interview-based qualitative study found that domains such as pain, walking and fear of complications, awareness of the artificial knee joint and return to work were important to people were important when recovering from a TKR. The two issues that matter across all participants was immediate and long-term postoperative pain and the ability to walk. Clinicians should consider these when informing potential patients about to undergo surgery, to aid informed and shared decision-making. Researchers should evaluate these domains to gain a richer and more complete picture of outcomes in patients within interventional studies.

## supplementary material

10.1136/bmjopen-2023-080795 online supplemental file 1

10.1136/bmjopen-2023-080795 online supplemental file 2

## Data Availability

All data relevant to the study are included in the article or uploaded as online supplemental information.
