# Supplementary material for: What recovery domains are important following a total knee replacement? A qualitative, interview-based study
Source: BMJ Open. 2024 May 9;14(5):e080795. doi: 10.1136/bmjopen-2023-080795 (PMC11086519; doi:10.1136/bmjopen-2023-080795)
Supplement: online supplemental file 1 [file bmjopen-14-5-s001.pdf]

Group I: Pre-operative population

Hello, my name is Chetan, I'm a doctor and researcher from Warwick University. I am conducting a research study to find out what factors people think are important when recovering from a knee replacement.

You have already been given an information sheet, the opportunity to ask questions and to sign a consent form specific to this interview study. I would also like to remind you that you can withdraw from this study at any time during the interview and up to 2 weeks after the completion of this interview.

During this interview I would like to ask how you expect your post-operative recovery to be like. I know you haven't had your operation yet. However, I would like to understand what factors you think are important after you have had your operation.

It is important to note that there is no right or wrong answers here, we are just exploring your opinions.

Can you please confirm for your recording that you understand this and are willing to continue?

Case vignette

Sometimes it is helpful to start with by considering a situation.

A 65-year-old woman is having a knee replacement for osteoarthritis of her knee. She wonders what her recovery after her operation looks like.

<u>Fixed questions</u>

1. **<u>General</u>**

- Can you tell me about the reasons you are considering a knee replacement?

- Is there anything you're worried about?

- What are you hoping your operation will achieve?

<u>Flexible questions</u>

2. **<u>Pain</u>**

- Some people have pain as a result of the arthritis of the knee. Does this affect you?
    - o How severe is your pain?
    - o Does it limit you in any way?

- Does it matter what time of day the pain is worst?
    - o How important is day-time pain?
    - o How important is night-time pain?
- What do you expect to happen to your pain after surgery?
- How important is the pain immediately after the operation to you (initial 6 weeks)? Sometimes this is called acute pain.
- How important is pain weeks to months after the operation to you (9-12 months)? Sometimes this is called chronic pain.
- Is there any area of pain after your operation that you think we haven't discussed and is important?

3. **<u>Function</u>**

In the next section I will ask about how your knee arthritis has affected your life in day-to-day activities.

- What function do you hope to gain after your knee operation?
- Could you tell me what the impact of arthritis of the knee has been on different aspects of your life?
    - What has been the impact on your daily life?
    - Has your arthritis affected your family life?
    - What has been the impact on your work/studies?
    - Did it affect your ability to play sport?
- How important is going on walks?
    - On flat surfaces or up and down hills?
    - For short walks or long walks?
    - Is it more important immediately after surgery (initial 6 weeks) or later on after surgery (9-12 months)
- How important is your ability to climb up or come down stairs?
    - Is it more important immediately after surgery (initial 6 weeks) or later on after surgery (9-12 months)
- How important is kneeling for you?
    - Is it more important immediately after surgery (initial 6 weeks) or later on after surgery (9-12 months)
- How important is it doing your favourite sport?
    - Is it more important immediately after surgery (initial 6 weeks) or later on after surgery (9-12 months)
- How would you feel if you couldn't get back to your favourite sport after your knee replacement?
- Is there any aspect of activities or function of your knee that we haven't discussed and you think is important to discuss?

4. Complications after the operation

- There are possible complications of having a total knee replacement. Are you aware of these (I can remind you)?
    - Which ones do you think are most important?
    - Do you think the risk of bleeding is important?
    - Do you think the risk of infection is important?
    - Do you think the risk of re-operation in the weeks or months after the first operation is important? This is usually due to complications such as infection
    - Do you think the risk of re-operation years after the first operation is important? This is often referred to risk of revision surgery for complications such as infection, loosening of implant or ongoing pain
- Are there any complications or discussion about complications after your operation that we haven't discussed and you think is important to?

5. Awareness of joint

- Some people are aware of their knee implant after their knee replacement, how important is this to you?
    - Is it more important immediately after surgery (initial 6 weeks) or later on after surgery (9-12 months)

<u>Closing comments</u>

- Is there anything else you would like to comment on regarding the things we have discussed?

Thank you for taking the time to share your views with me, I really appreciate it. Please do contact me if you have any questions regarding the study. My contact details are found on the patient information sheet.

Group II: Post-operative population

Hello, my name is Chetan, I'm a researcher from Warwick University.

I am conducting a research study to find out what factors people think are important when recovering from a knee replacement.

You have already been given an information sheet, the opportunity to ask questions and to sign a consent form specific to this interview study. I would also like to remind you that you can withdraw from this study at any time during the interview and up to 2 weeks after the completion of this interview. You don't have to give me a reason why

During this interview I would like to ask you what mattered to you when you were recovering from your knee operation.

It is important to note that there are no right or wrong answers here, I'm just exploring your opinions and what was important for you.

Can you please confirm for your recording that you understand this and are willing to continue?

Case vignette

Sometimes it is helpful to start with a situation.

A 65-year-old woman is having a knee replacement for osteoarthritis of her knee. She wonders what her recovery after her operation looks like

Fixed questions

1. **General**

- Can you tell me how your operation went?
- How has your recovery been?
- What have been the benefits to you since having the operation?
- Did you encounter any problems since you had the surgery?

Flexible questions

2. **Pain**

- Some people have pain as a result of arthritis of the knee. Did this affect you?
    - How severe was your pain?
    - Did it limit you in any way?
- Does it matter what time of day the pain is worst?
    - How important is day time pain?
    - How important is night time pain?
    - Which was more important, the day or night-time pain?
- What did you expect to happen to your pain after surgery?
- How important was the pain immediately after the operation to you (initial 6 weeks)? Sometimes this is called acute pain.
- How important was pain weeks to months after the operation to you (9-12 months)? Sometimes this is called chronic pain

- Is there any area of pain after your operation that you think we haven’t discussed and is important?

3. **Function**

- What function did you hope you gain after your operation?
- Could you tell me what the impact of your knee replacement has been on different aspects of your life?
  - What has been the impact on your daily life?
  - Has your knee affected your family life? Tell me more?
  - What has been the impact on your work/studies?
  - Did it affect your ability to play sport? Could you tell me more?
- How important is going on walks?
  - On flat surfaces or up and down hills?
    For short walks or long walks?
  - Is it more important immediately after surgery (initial 6 weeks) or later on after surgery (9-12 months)
- How important is your ability to climb or come down stairs?
  - Is it more important immediately after surgery (initial 6 weeks) or later on after surgery (9-12 months)
- How important is kneeling for you?
  - Is it more important immediately after surgery (initial 6 weeks) or later on after surgery (9-12 months)
- How important is it doing your favourite sport?
  - Is it more important immediately after surgery (initial 6 weeks) or later on after surgery (9-12 months)
- How would you feel if you couldn’t get back to your favourite sport after your knee replacement?
- Is there any aspect of activities or function of your knee that we haven’t discussed and you think is important to discuss?

4. **Complications after the operation**

- There are possible complications of having a total knee replacement. Are you aware of these (I can remind you)?
  - Which ones do you think are most important?
  - Do you think the risk of bleeding is important?
  - Do you think the risk of infection is important?
  - Do you think the risk of re-operation in the weeks or months after the first operation is important? This is usually due to complications such as infection
  - Do you think the risk of re-operation years after the first operation is important? This is often referred to risk of revision surgery for complications such as infection, loosening of implant or on-going pain
- Are there any complications or discussion about complications after your operation that we haven’t discussed and you think is important to?

5. Awareness of joint

- Some people are aware of their artificial knee after their knee replacement, how important is this to you?
  - Is it more important immediately after surgery (initial 6 weeks) or later on after surgery (9-12 months)

<u>Closing comments</u>

- Is there anything else you would like to comment on regarding the things we have discussed?

Thank you for taking the time to share your views with me, I really appreciate it. Please do contact me if you have any questions regarding the study. My contact details are found on the patient information sheet.
