# Supplementary material for: What recovery domains are important following a total knee replacement? A qualitative, interview-based study
Source: BMJ Open. 2024 May 9;14(5):e080795. doi: 10.1136/bmjopen-2023-080795 (PMC11086519; doi:10.1136/bmjopen-2023-080795)
Supplement: online supplemental file 2 [file bmjopen-14-5-s002.pdf]

Supplementary file 2: Further quotes per theme

Pain

Pain subtheme: Early vs Late pain

P4 (60M LATE POST-OPERATIVE): *I'd like to continue improving the way I am because I believe that I am prove I am improving week by week to be quite honest with you.*

P5 (61F LATE-POSTOPERATIVE): *I tell you what, the day after when I woke up. I couldn't believe it. It was the pain from the operation was there. But the other pain had gone.*
P5 (61F LATE-POSTOPERATIVE): *It was really bad when I got home. Thing is, you get the morphine and all that in the hospital, but you don't get much of it to go hone with. And then I didn't know whether it was normal … or who I could go get some tablets from.*
P5 (61F LATE-POSTOPERATIVE): *I don't want long term pain. No, I'd rather have that short term pain.*

P7 (53F EARLY POST-OPERATIVE): *Yeah, well, I thought, well, it well. It's a major operation, isn't it? So I was expecting some sort of like, you know, discomfort and pain and because you've gotta get it moving back again, don't you? …*

Interviewer: What do you expect the pain to be like in about a years time?
P7 (53F EARLY POST-OPERATIVE): *No pain free preferably.* …
P7 (53F EARLY POST-OPERATIVE): *The discomfort is fine because I know sooner or later it will calm down*

P9 (67F PRE-OPERATIVE): *I am worried about post-op pain cause that was really painful.* …
P9 (67F PRE-OPERATIVE): *Yeah, that was for a couple of weeks. It was really. You know, really bad. I did wish that I hadn't had it done …*
P9 (67F PRE-OPERATIVE): *So I'm thinking afterward money up is gonna be painful for a month or so, but then that's only short term, and then it's gonna get better.*

Pain subtheme: Day vs Night-time pain

P2 (65M EARLY POST-OP): *Yeah, the daytime that hurt me more than in the evening.*

Interviewer: Did it matter what time of day?
P3 (53M PRE-OPERATIVE): *No, not really. It's all there's there at night when I turn in the bed, you know, I can feel it so. It's always a constant. Before it used to bother me just at night, now it's always there.*

P4 (60M LATE POST-OPERATIVE): *I would say daytime really because there's more active in the day and I notice the pay more in the day*

Pain subtheme: Medication to relieve pain

Interviewer: Is it important for you not to take pain tablets?
P8 (51F EARLY POST-OPERATIVE): *I'm not a tablet person and I'd rather the operation get rid of the pain so I wouldn't have to.*

P5 (61F LATE-POSTOPERATIVE): *Even if they're prescribed by people I, you know, by medical profession, I'm I'd rather not take them to be fair.*

Function

Function subtheme: Ability to walk

P8 (51F EARLY POST-OPERATIVE): *Obviously, I'm a police officer, so I was removed off sort of uniform duty to desk duties and. Just couldn't walk more than sort of more than 10 minutes without severe pain in the knee, so it got to the point where it was getting more desperate than anything.*

Interview: what were you hoping that the operation would do for you?
P1 (62M LATE POST-OP): *Mainly walks. I'd like to play I'd like to be able to walk around a golf course again, if possible, but that might not happen. So I can't really walk that far because I've got arthritis among other knee. So it's probably that's the reason.*

Interviewer: can you tell me about some of the reasons why you considered having a new replacement?
P6 (78F PRE-OPERATIVE): *So that I could walk again, really without pain.*

Function subtheme: Ability to Kneel

P2 (65M EARLY POST-OP): *I do a lot of kneeling because I do a lot of my own work on the farm, but we've got I tend to keep some old cushions about what's probably chucked out from the old settees. And so I kneel on cushions because I can't kneel on the concrete and tarmac.*

P9 (67F PRE-OPERATIVE): *Somebody said that you're not supposed to need it when you've had a knee replacement. That it could damage it. I don't know if that's true.*

Function subtheme: Normal activities in the home

P2 (65M EARLY POST-OP): *I couldn't quite tackle the stairs. That's why they let me at the hospital because I'm my upper body was very strong. I could tackle the stairs. But when I got home, I thought I'll stay downstairs for a couple of weeks just to be on the same side*

P2 (65M EARLY POST-OP): *You know, I couldn't get out of the chair. And sometimes I have to get my wife to help me out the chair because my leg seemed to have set and stiffened*.

P3 (53M PRE-OPERATIVE): *I expect that I will have full movement up being able to go up and down the stairs without a problem at all. I think the surface of my knee will be perfectly aligned with metal plastic. I'm expecting that. I don't want to have the unevenness way of my knee, so badly worn that it will work mechanically as it's as it should do.*

Function subtheme: Flexibility and range of motion of knee joint

P8 (51F EARLY POST-OPERATIVE): *The flexibility it's the, the bend, the easiness of bending getting upstairs as I normally would.*

P8 (51F EARLY POST-OPERATIVE): *The knees not completely straight yet it possible I'd get there. It makes going down the stairs hard.*

Function subtheme: Sport

P1 (62M LATE POST-OP): *I used to play golf after the football. Yeah, quite a number of years. But then it got gradually worse and worse. I couldn't even more on the golf course anymore. You can only walk about 200 yards. So actually, if I did play golf, I had to go on a buggy and just get off and on, take the shot and get without the walking part of it. …*
I'd like to play I'd like to be able to walk around a golf course again, once my operation makes my knee better

P4 (60M LATE POST-OPERATIVE): *The action of kicking a ball a ball can be quite heavy, and now you're kicking a football with a knee that's been replaced. I think you can actually damage your knee or you can cause a fair bit of pain.*

P9 (67F PRE-OPERATIVE): *No, no, because I've I mean, obviously, when the children were younger. We played football, cricket, whatever. But not anymore, no. It isn't relevant because I don't do any (sport).*

Fear of Complications

Interviewer: Do you think the risk of bleeding is important after the operation?
P1 (62M LATE POST-OP): *Not really. No, Not really. Fine.*

P11 (73F EARLY POST-OPERATIVE): *And you think ohh and that, but then you know you could get run over by a bus, couldn't you? So that they would just general.*

P3 (53M PRE-OPERATIVE): *I guess I'm less worried that is it's a concern, but you know that everything's been done to make sure that the environment that we're having that surgery is clean, that all the tools are there, everybody's scrubbed up properly. So I I'm less it's a worry, but you know it's a bit of a numbers game.*

P10 (63F PRE-OPERATIVE): *I think infection scares me most because of the potential consequences of getting an infection in a knee replacement.*

P10 (63F PRE-OPERATIVE): *I think avoiding infection is important to avoid another operation. Because it just categorized think as major surgery, isn't it? So it you know, you wouldn't want to have major surgery unnecessarily. It shouldn't be considered. You shouldn't enter into it lightly.*

So that's hugely important. When I was kind of worried in the early stages, thinking is this pain normal or something gone wrong? If I got an infection, the thought of somebody actually opening that wound up on that leg was just absolutely. Terrifying, you know.

P2 (65M EARLY POST-OP): *Yeah, it was because I did. I did suffer from blood clots, and that was one of my big concerns with the operation. I mean, because I suffered from blood clots.*

P4 (60M LATE POST-OPERATIVE): *Now it was it was suspected DVT. It wasn't actually confirmed as DVT as such, but I did actually undergo treatment for it. A clot, if you like. So I had to go on to some medication, blood thinning medication, et cetera. That cleared up pretty quickly really. But it won't. It was a concern at the time because it was very, very painful. My, my leg was very well, very sore, I couldn’t move properly cause of the pain, you know. And did the pain mean something was wrong in my knee joint?*

Ability to forget artificial knee joint

P5 (61F LATE-POSTOPERATIVE): *Yeah, it is. Yeah. Because I don't want to be thinking about that all the time. No. So it's important to me to me, just to carry on*

P9 (67F PRE-OPERATIVE): *I don't mind being aware of it. You know, like the second part of your what you was asking. Don't think I'd want it that I'm not aware of it at all because. Maybe that's not safe.*

P3 (53M PRE-OPERATIVE): *I honestly don't know what that means. I don't know how to answer that. I'm sorry, I guess.*

Return to work

P9 (67F PRE-OPERATIVE): *At this in a disheartening really, isn't it? When you can't do things. I want to be able to do my job properly.*
