# Supplementary material for: What recovery domains are important following a total knee replacement? A qualitative, interview-based study
Source: BMJ Open. 2024 May 9;14(5):e080795. doi: 10.1136/bmjopen-2023-080795 (PMC11086519; doi:10.1136/bmjopen-2023-080795)
Supplement: Supplementary file 3 [file bmjopen-2023-080795.reviewer_comments.pdf]

PEER REVIEW HISTORY

BMJ Open publishes all reviews undertaken for accepted manuscripts. Reviewers are asked to complete a checklist review form (**http://bmjopen.bmj.com/site/about/resources/checklist.pdf**) and are provided with free text boxes to elaborate on their assessment. These free text comments are reproduced below.

ARTICLE DETAILS

**TITLE (PROVISIONAL)**	What recovery domains are important following a total knee replacement?: A qualitative, interview based study
**AUTHORS**	Khatri, Chetan; Dhaif, Fatema; Ellard, David; Rodrigues, Jeremy; Underwood, Martin; Mitchell, Paul; Metcalfe, Andrew

VERSION 1 – REVIEW

**REVIEWER**	van den Ende, Cornelia<br>Sint Maartenskliniek, Department of Rheumatology
**REVIEW RETURNED**	03-Nov-2023

**GENERAL COMMENTS**	This manuscript reports on the results of a qualitative study among pre- and postoperative patients about their experiences after total knee replacement.<br>My main concern is that the study looks more like a survey with open-ended questions rather than a qualitative study. Authors suggest that themes emerged through analysis of the interviews. In fact, the interview guide consisting of explicit questions about different outcomes introduced these themes. Many questions of the interview guide were quite leading; e.g. “How important is kneeling for you? And “Is it more important immediately after surgery (initial 6 weeks) or later on after surgery (9-12 months?”.<br>The introduction lacks an overview of results of previous studies on this subject. In line with this: the discussion lacks a reflection on the extent to which this study contributes to the existing body of knowledge.<br>It seems to me that the research question “What is important to people when recovering from a total knee replacement” implies that the process of recovery is the leading question. However, both the interview guide and results section emphasise the outcome of total knee replacement, rather than the process itself.<br>Important information in the method section is lacking about the following aspects:<br>- Recruitment of patients. How and by whom were patients selected? Who informed and approached participants?<br>- Why was a case vignette used? Of note: the case vignette was not included in the supplementary file.<br>- Were there any patients approached treated by one of the authors?<br>- More detail of the different steps of coding is needed. A code tree is lacking<br>I could not trace the systematic review upon the interview guide was based, ref 17<br>The length of interviews seems rather short, half of them lasted less than 30 minutes. This questions the likelihood that patients were really given the opportunity to tell their own story.

	P 8 L 30-32: I do not understand why 25% of the interviews were coded by two reviewers to ensure saturation?<br>In the results section themes are presented and followed by discussion. Please, separate the results and discussion section.<br>Experiences of pre-operative patients are presented without any connection with the research question.<br>The discussion section lacks a summary of the results.<br>Three of the four strengths and limitations address general issues related to qualitative research but do not address issues of the presented study itself.

**REVIEWER**	Goh, En Lin<br>University of Oxford, Oxford University Clinical Academic Graduate School
**REVIEW RETURNED**	19-Dec-2023

**GENERAL COMMENTS**	This piece of qualitative research provides useful information and insight into the perspective of patient's undergoing a total knee replacement. I have several comments:<br>1. Were there any differences in themes/subthemes between patients interviewed in the pre-op, early post-op and late post-op phases? For example, was there less focus on pain in patients pre-op compared to those post-op?<br>2. Please re-structure the manuscript to separate the results and discussion for the themes and subthemes.

**REVIEWER**	Claireaux, Henry<br>University of Oxford, Nuffield Department of Orthopaedics, Rheumatology and Musculoskeletal Sciences
**REVIEW RETURNED**	11-Jan-2024

**GENERAL COMMENTS**	This is a well-conducted and written-up qualitative study. The abstract is a clear distillation of the remainder of the paper. The background information in the introduction is appropriate. The methods are clear and would allow the reproduction of the study by another group. The results are clearly laid out. Due to the nature of the study a statistical review is not recommended. The data presented support the authors conclusions as sated in the discussion. I have no concerns about the standard of English.

VERSION 1 – AUTHOR RESPONSE

**Reviewer 1: Dr. Cornelia van den Ende, Sint Maartenskliniek**	
My main concern is that the study looks more like a survey with open-<br>ended questions rather than a qualitative study. Authors suggest that themes emerged through analysis of the interviews. In fact, the interview guide consisting of explicit questions about different outcomes introduced these themes. Many questions	Thank you, we appreciate it may be misleading that the topic guide included direct questions rather than open ended questions and recognise the importance of these comments from the reviewer.

of the interview guide were quite leading; e.g. "How important is kneeling for you? And "Is it more important immediately after surgery (initial 6 weeks) or later on after surgery (9-12 months?".	As an explanation:<br><br>The topic guide was adapted as an iterative process throughout interviews, based on the constant comparative analysis approach we undertook. The presented topic guide is the final version, which was compiled at the end of the interview schedule.<br><br>Based on participant feedback prior to starting this work, we started interviews with an introduction of the purpose of the interviews. We included a case vignette to frame and contextualise the interview.<br><br>We subsequently followed these up with three general questions (in the pre-operative population):<br><br>- Can you tell me about the reasons you are considering a knee replacement?<br>- Is there anything you're worried about?<br>- What are you hoping your operation will achieve?<br><br>These questions were designed to be open ended questions, which were intended as a starting point. Topics of conversation were built organically from these. For example, if participants replied to the open-ended questions 'what are you hoping your operation will achieve?' with 'I am hoping the operation will decrease the pain I am in', a conversation exploring pain would then take place.<br><br>These three questions represent the only questions that were scripted for the first interview. Based on analysis from the first interview (and then subsequent interviews), the topic guide was iteratively developed.<br><br>The closed, direct questions about each domain were included to ensure topics that were identified by participants were discussed with subsequent, future participants. These were categorised under pain, function, complications after the operation and awareness of joint. These closely mirror the final five themes and are reflective of the constant-comparative analysis we undertook.

	Direct questions were not always posed to the participants, as long as participants brought up the themes in their discussions.<br><br>We have clarified this in our methods and have clarified the topic guide to reflect this. In addition, we have included a “Box 1” which lists the open-ended questions used at the start of every interview.
The introduction lacks an overview of results of previous studies on this subject. In line with this: the discussion lacks a reflection on the extent to which this study contributes to the existing body of knowledge.	Thank you, we have expanded the introduction by highlighting results from previous studies to demonstrate the need for our presented manuscript.<br><br>We have also expanded sections in the discussion to compare to existing literature.
It seems to me that the research question “What is important to people when recovering from a total knee replacement” implies that the process of recovery is the leading question. However, both the interview guide and results section emphasise the outcome of total knee replacement, rather than the process itself.	Thank you, we did wish to focus on the recovery after total knee replacement, referring to the post-operative phase, and not the operation itself.<br><br>Other authors have sought to explore the process of TKR. We sought to focus on the recovery phase can be measured by various outcomes, such as pain, function, or satisfaction.<br><br>We have expanded the background to clarify this and provide a brief overview of the current literature into this topic.
Recruitment of patients. How and by whom were patients selected? Who informed and approached participants?	Thank you, I have included this as a sub-section ‘Recruitment’ under methods.
Why was a case vignette used? Of note: the case vignette was not included in the supplementary file.	Thank you, we have expanded the methods discussing topic guide and the advantages using a case vignette provides. This case vignette was developed and checked with participant-partners when designing the study.

	We have included the case vignette in the topic guide.
Were there any patients approached treated by one of the authors?	Thank you. This is an important point to clarify. None of the participants were in the direct treatment by any of the authors. We have detailed this in the methods.
More detail of the different steps of coding is needed. A code tree is lacking	Thank you, we have included a code tree as a figure.
I could not trace the systematic review upon the interview guide was based, ref 17	Thank you, we apologise for this as the publication was in press at the time of submission. We have updated this in the manuscript and the review is available at:<br><br>Khatri C, Ahmed I, Dhaif F, et al. What's important for recovery after a total knee replacement? A systematic review of mixed methods studies. *Archives of Orthopaedic and Trauma Surgery* 2023:1-9.
The length of interviews seems rather short, half of them lasted less than 30 minutes. This questions the likelihood that patients were really given the opportunity to tell their own story.	Thank you, we note the mean interview length of all interviews was 27.5 minutes.<br><br>Nevertheless, we appreciate the comment from the reviewer. We have re-reviewed the transcripts relating to the interviews that lasted less than 20 minutes, with consideration and critical thought whether participants were allowed to tell their own story.<br><br>Upon review of the manuscripts, we noted that all participants stated that their post-operative pain was more than they had expected, insinuating they were currently in pain. Although, as we did not collect any pain scores or any other quantitative PROM data, we are unable to ascertain this.<br><br>We postulate whether the length of interview was influenced by this and have included this in the strengths and limitations.

P 8 L 30-32: I do not understand why 25% of the interviews were coded by two reviewers to ensure saturation?	Thank you, by using double coding, we were able to ensure that any potential biases from the lead researcher were minimised. It allowed for true thematic saturation to be reached, rather than just perceived to have been by one researcher.<br><br>Two researchers coding also supplemented and facilitated contesting views and opinions and therefore enhancing reflexivity.<br><br>We have expanded this in the methods section, and this is also discussed in the strengths and limitations.
In the results section themes are presented and followed by discussion. Please, separate the results and discussion section.	Thank you, given the strong steer by the editor, and two out of three reviewers, we have reverted to a traditional structure.
Experiences of pre-operative patients are presented without any connection with the research question.	Thank you, we have clarified and expanded this in the results and discussion.
The discussion section lacks a summary of the results.	Thank you, this was not included in the previous draft due to the structure of a combined results and discussion.<br><br>In the new structure, we have structured the discussion to have a summary of the results, followed by discussion about individual domains and comparison to the literature, followed by strengths and limitations.
Three of the four strengths and limitations address general issues related to qualitative research but do not address issues of the presented study itself.	Thank you, we have contextualised issues highlighted in the strengths and limitations section to relate to the presented manuscript.
**Reviewer 2: Dr. En Lin Goh, University of Oxford**	

This piece of qualitative research provides useful information and insight into the perspective of patient's undergoing a total knee replacement	Thank you.
Were there any differences in themes/subthemes between patients interviewed in the pre-op, early post-op and late post-op phases? For example, was there less focus on pain in patients pre-op compared to those post-op?	Thank you, this is an interesting thought.<br><br>We did not explore themes by sub-population and believe our current study design is underpowered to fully appreciate differences between participants at different operative states.<br><br>Interviews were terminated when thematic saturation was reach amongst the sampled study population. To effectively explore a research question of whether themes differed according to operative status, we would consider extending our interview schedule and analysing according to operative status, until we achieve thematic saturation in each sub-population.<br><br>As such, this represents an interesting research question and the topic for future research. Unfortunately, due to resource limitations, we are unable to include this in the present manuscript. We have included this in our discussion as future direction.<br><br>Of note, this interview schedule has formed the basis of health preferencing work (discrete choice experiment). Participants can trade recovery domains such as pain and function to establish relative importance of individual domains. Within this, we can explore in subpopulations, and this could be used to answer the research question of whether pain is more (or less) important to participants who are pre-operative, when compared to participants who are post-operative.
Please re-structure the manuscript to separate the results and discussion for the themes and subthemes.	Thank you, we had combined results and discussion by theme as we perceived discussion of individual themes in context.

	However, given the strong steer by the editor, and two reviewers, we have reverted to a traditional structure.
**Reviewer 3: Mr. Henry Claireaux, University of Oxford**	
This is a well-conducted and written-up qualitative study. The abstract is a clear distillation of the remainder of the paper. The background information in the introduction is appropriate. The methods are clear and would allow the reproduction of the study by another group. The results are clearly laid out.	Thank you.
Due to the nature of the study a statistical review is not recommended.	Thank you.
The data presented support the authors conclusions as sated in the discussion. I have no concerns about the standard of English.	Thank you.
